# Supplementary material for: Effects of General Physical Activity Promoting Interventions on Functional Outcomes in Patients Hospitalized over 48 Hours: A Systematic Review and Meta-Analysis of Randomized Controlled Trials
Source: Int J Environ Res Public Health. 2021 Jan 29;18(3):1233. doi: 10.3390/ijerph18031233 (PMC7908565; doi:10.3390/ijerph18031233)
Supplement: Supplementary file 1 [file ijerph-18-01233-s001.zip › Supplementary material.docx]

**Supplementary material**

***Table S1****. Complete search strategy for MEDLINE, searched from inception till January 2020.*

| ((((randomized controlled trial[pt] OR controlled clinical trial[pt] OR randomized[tiab] OR placebo[tiab] OR clinical trials as topic[mesh:noexp] OR randomly[tiab] OR trial[ti] NOT (animals[mh] NOT humans[mh])))) |
| --- |
| **AND** (("Inpatients"[Mesh] OR "Hospitalization"[Mesh:noexp] OR inpatients[tiab] OR inpatient[tiab] OR hospitalized[tiab] OR hospitalization[tiab] OR hospitalizations[tiab] OR hospitalised[tiab] OR hospitalisation[tiab] OR hospitalisations[tiab] OR hospital[tiab]))) |
| **AND** (("Physical Therapy Modalities"[Mesh] OR "Early Ambulation"[Mesh] OR "Exercise"[Mesh] OR "Physical Fitness"[Mesh] OR "Exercise Therapy"[Mesh] OR "Early Medical Intervention"[Mesh] OR "Locomotion"[Mesh] OR "Self Care"[Mesh] OR Cardiorespiratory Fitness[tiab] OR Early Medical Intervention*[tiab] OR Locomotion[tiab] OR mobility[tiab] OR mobile intervention*[tiab] OR physical fitness[tiab] OR Physical therapy[tiab] OR Physiotherapy[tiab] OR Physical Activity[tiab] OR Physical Activities[tiab] OR Physical activation[tiab] OR stepping[tiab] OR (Sedentary lifestyle[mesh] OR Sedentary lifestyle[tiab] OR Sedentary behaviour[tiab] OR Sedentary behavior[tiab] OR Physical inactivity[tiab] OR Immobility[tiab] OR Immobilization syndrome*[tiab] OR Iatrogenic disability[tiab]))) |
| **AND** (("Activities of Daily Living"[Mesh] OR "Length of Stay"[Mesh] OR Muscle strength[MeSH] OR "Patient Readmission"[Mesh] OR "Patient Discharge"[Mesh] OR "Quality of Life"[Mesh] OR "Outcome Assessment Health Care"[Mesh:noexp] OR "Patient Outcome Assessment"[Mesh:noexp] OR "Patient Reported Outcome Measures"[Mesh] OR "Treatment Outcome"[Mesh] OR "Recovery of Function"[Mesh] OR Functional outcome[tiab] OR Functional outcomes[tiab] OR Functional recovery[tiab] OR Functional impairment[tiab] OR Functional decline[tiab] OR Functional loss[tiab] OR Disability[tiab] OR Disabilities[tiab] OR Inability[tiab] OR Inabilities[tiab] OR Muscle strength[tiab] OR Length of Stay[tiab] OR Stay Length[tiab] OR Stay Lengths[tiab] OR Hospital Stay[tiab] OR Hospital Stays[tiab] OR Readmission[tiab] OR Readmissions[tiab] OR Patient Discharge[tiab] OR Patient Discharges[tiab] OR ADL[tiab] OR Activities of daily living[tiab] OR Daily Living Activity[tiab] OR Daily Living Activities[tiab] OR Life Quality[tiab] OR Quality of life[tiab] OR Patient Reported Outcome[tiab] OR Patient Reported Outcomes[tiab] OR Treatment outcome[tiab] OR Treatment outcomes[tiab] OR Clinical Effectiveness[tiab] OR Clinical Effectivenesses[tiab] OR Clinical Efficacy[tiab] OR Patient Relevant Outcome[tiab] OR Patient Relevant Outcomes[tiab] OR Treatment Effectiveness[tiab] OR Treatment Effectivenesses[tiab] OR Treatment Efficacy[tiab] OR Rehabilitation Outcome[tiab] OR Rehabilitation Outcomes[tiab] OR Patient Outcome[tiab] OR Patient Outcomes[tiab])) |

**
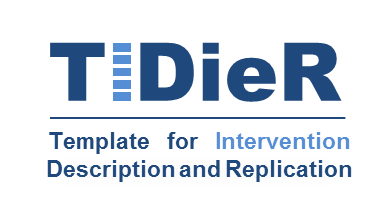
The TIDieR (Template for Intervention Description and Replication) Checklist*:**

Information to include when describing an intervention and the location of the information

***Table S2.*** *Study: Brown CJ, Foley KT, Lowman JD, Jr., et al. Comparison of posthospitalization function and community mobility in hospital mobility program and usual care patients: a randomized clinical trial. JAMA Itern Med 2016;176:921-7.*

| **Item number** | **Item** | **Where located **** |
| --- | --- | --- |
|  |  | Primary paper (page or appendix number) |
|  | **BRIEF NAME** |  |
| **1.** | Provide the name or a phrase that describes the intervention. | Hospital mobility program (MP) (p.923)  Not described  Hopital MP Gait belts were used to ensure safe ambulation (p. 923)  To assist in self-monitoring of out-of-bed mobility, the MP patients were provided with a diary that could be used to document each time they sat up or walked. (p. 924)  Assistance to walking with behavioural intervention integration (p. 923-924)  Research team members, training by BVAMC physical therapists (p. 923)  Face to face, individual (p. 923-924)  In-hospital (p. 923)  Twice a day for 15-20 minutes each session 7 days a week (p. 923)  Not described  Not described  Not described  The mobility program group completed 122 of the potential 238 walks (51%) and 108 of the 135 visits (80%) (p. 924-925) |
|  | **WHY** |  |
| **2.** | Describe any rationale, theory, or goal of the elements essential to the intervention. |  |
|  | **WHAT** |  |
| **3.** | Materials: Describe any physical or informational materials used in the intervention, including those provided to participants or used in intervention delivery or in training of intervention providers. Provide information on where the materials can be accessed (e.g. online appendix, URL). |  |
| **4.** | Procedures: Describe each of the procedures, activities, and/or processes used in the intervention, including any enabling or support activities. |  |
|  | **WHO PROVIDED** |  |
| **5.** | For each category of intervention provider (e.g. psychologist, nursing assistant), describe their expertise, background and any specific training given. |  |
|  | **HOW** |  |
| **6.** | Describe the modes of delivery (e.g. face-to-face or by some other mechanism, such as internet or telephone) of the intervention and whether it was provided individually or in a group. |  |
|  | **WHERE** |  |
| **7.** | Describe the type(s) of location(s) where the intervention occurred, including any necessary infrastructure or relevant features. |  |
|  | **WHEN and HOW MUCH** |  |
| **8.** | Describe the number of times the intervention was delivered and over what period of time including the number of sessions, their schedule, and their duration, intensity or dose. |  |
|  | **TAILORING** |  |
| **9.** | If the intervention was planned to be personalised, titrated or adapted, then describe what, why, when, and how. |  |
|  | **MODIFICATIONS** |  |
| **10.^ǂ^** | If the intervention was modified during the course of the study, describe the changes (what, why, when, and how). |  |
|  | **HOW WELL** |  |
| **11.** | Planned: If intervention adherence or fidelity was assessed, describe how and by whom, and if any strategies were used to maintain or improve fidelity, describe them. |  |
| **12.^ǂ^** | Actual: If intervention adherence or fidelity was assessed, describe the extent to which the intervention was delivered as planned. |  |

** **Authors** - use N/A if an item is not applicable for the intervention being described. **Reviewers** – use ‘?’ if information about the element is not reported/not sufficiently reported.

† If the information is not provided in the primary paper, give details of where this information is available. This may include locations such as a published protocol or other published papers (provide citation details) or a website (provide the URL).

ǂ If completing the TIDieR checklist for a protocol, these items are not relevant to the protocol and cannot be described until the study is complete.

* We strongly recommend using this checklist in conjunction with the TIDieR guide (see *BMJ* 2014;348:g1687) which contains an explanation and elaboration for each item.

**
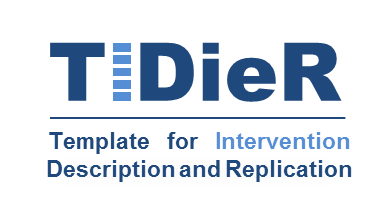
The TIDieR (Template for Intervention Description and Replication) Checklist*:**

Information to include when describing an intervention and the location of the information

***Table S3.*** *Study: Dall CH, Andersen H, Povlsen TM, Henriksen M. Evaluation of a technology assisted physical activity intervention among hospitalised patients: A randomised study. European journal of internal medicine. 2019;69:50-6.*

| **Item number** | **Item** | **Where located **** |
| --- | --- | --- |
|  |  | Primary paper (page or appendix number) |
|  | **BRIEF NAME** |  |
| **1.** | Provide the name or a phrase that describes the intervention. | Visual feedback (VF) (p. 52)  Not described  Visual feedback was provided with a tablet placed on the bedside table (p. 52-54)  The display showed current day’s activities and ‘smiley faces’, depending on time spent active or inactive (p. 54)  The physiotherapists mounted the accelerometers, and if appropriate according to allocation, installed feedback screens (7 in. tablets) on the bedside table (p. 54)  Via display, individual (p. 54)  In-hospital (p. 52-54)  Continuously, updated every 5 minutes 7 days a week (p. 54)  Tailored to basic mobility score, subgroups: CAS walking 1 or CAS walking 2  Not described  Not described  Not described |
|  | **WHY** |  |
| **2.** | Describe any rationale, theory, or goal of the elements essential to the intervention. |  |
|  | **WHAT** |  |
| **3.** | Materials: Describe any physical or informational materials used in the intervention, including those provided to participants or used in intervention delivery or in training of intervention providers. Provide information on where the materials can be accessed (e.g. online appendix, URL). |  |
| **4.** | Procedures: Describe each of the procedures, activities, and/or processes used in the intervention, including any enabling or support activities. |  |
|  | **WHO PROVIDED** |  |
| **5.** | For each category of intervention provider (e.g. psychologist, nursing assistant), describe their expertise, background and any specific training given. |  |
|  | **HOW** |  |
| **6.** | Describe the modes of delivery (e.g. face-to-face or by some other mechanism, such as internet or telephone) of the intervention and whether it was provided individually or in a group. |  |
|  | **WHERE** |  |
| **7.** | Describe the type(s) of location(s) where the intervention occurred, including any necessary infrastructure or relevant features. |  |
|  | **WHEN and HOW MUCH** |  |
| **8.** | Describe the number of times the intervention was delivered and over what period of time including the number of sessions, their schedule, and their duration, intensity or dose. |  |
|  | **TAILORING** |  |
| **9.** | If the intervention was planned to be personalised, titrated or adapted, then describe what, why, when, and how. |  |
|  | **MODIFICATIONS** |  |
| **10.^ǂ^** | If the intervention was modified during the course of the study, describe the changes (what, why, when, and how). |  |
|  | **HOW WELL** |  |
| **11.** | Planned: If intervention adherence or fidelity was assessed, describe how and by whom, and if any strategies were used to maintain or improve fidelity, describe them. |  |
| **12.^ǂ^** | Actual: If intervention adherence or fidelity was assessed, describe the extent to which the intervention was delivered as planned. |  |

** **Authors** - use N/A if an item is not applicable for the intervention being described. **Reviewers** – use ‘?’ if information about the element is not reported/not sufficiently reported.

† If the information is not provided in the primary paper, give details of where this information is available. This may include locations such as a published protocol or other published papers (provide citation details) or a website (provide the URL).

ǂ If completing the TIDieR checklist for a protocol, these items are not relevant to the protocol and cannot be described until the study is complete.

* We strongly recommend using this checklist in conjunction with the TIDieR guide (see *BMJ* 2014;348:g1687) which contains an explanation and elaboration for each item.

***Table S4.*** *Study: Killey B, Watt E. The effect of extra walking on the mobility, independence and exercise self-efficacy of elderly hospital in-patients: a pilot study. Contemp Nurse 2006;22:120-33.*

| **Item number** | **Item** | **Where located **** |
| --- | --- | --- |
|  |  | Primary paper  (page or appendix number) |
|  | **BRIEF NAME** |  |
| **1.** | Provide the name or a phrase that describes the intervention. | Supervised or assisted walk (p. 125)  Walking improves gait, mobility and exercise self-efficacy (p. 121-124)  This consisted of providing a chair to rest on at their first request, and then after resting, walking back to the bed area (p. 125)  Supervised or assisted walking (p. 125)  Nursing staff (p. 125)  Face-to-face, individual (p. 125)  In-hospital (p. 125)  Twice a day, seven days a week, maximum distance able to be comfortably walked (p. 125)  Tailored according to the comfort of patients (p. 125)  Not described  Not described  In the intervention group, 27 out of 29 completed >70% of the twice daily walks (p. 127) |
|  | **WHY** |  |
| **2.** | Describe any rationale, theory, or goal of the elements essential to the intervention. |  |
|  | **WHAT** |  |
| **3.** | Materials: Describe any physical or informational materials used in the intervention, including those provided to participants or used in intervention delivery or in training of intervention providers. Provide information on where the materials can be accessed (e.g. online appendix, URL). |  |
| **4.** | Procedures: Describe each of the procedures, activities, and/or processes used in the intervention, including any enabling or support activities. |  |
|  | **WHO PROVIDED** |  |
| **5.** | For each category of intervention provider (e.g. psychologist, nursing assistant), describe their expertise, background and any specific training given. |  |
|  | **HOW** |  |
| **6.** | Describe the modes of delivery (e.g. face-to-face or by some other mechanism, such as internet or telephone) of the intervention and whether it was provided individually or in a group. |  |
|  | **WHERE** |  |
| **7.** | Describe the type(s) of location(s) where the intervention occurred, including any necessary infrastructure or relevant features. |  |
|  | **WHEN and HOW MUCH** |  |
| **8.** | Describe the number of times the intervention was delivered and over what period of time including the number of sessions, their schedule, and their duration, intensity or dose. |  |
|  | **TAILORING** |  |
| **9.** | If the intervention was planned to be personalised, titrated or adapted, then describe what, why, when, and how. |  |
|  | **MODIFICATIONS** |  |
| **10.^ǂ^** | If the intervention was modified during the course of the study, describe the changes (what, why, when, and how). |  |
|  | **HOW WELL** |  |
| **11.** | Planned: If intervention adherence or fidelity was assessed, describe how and by whom, and if any strategies were used to maintain or improve fidelity, describe them. |  |
| **12.^ǂ^** | Actual: If intervention adherence or fidelity was assessed, describe the extent to which the intervention was delivered as planned. |  |

** **Authors** - use N/A if an item is not applicable for the intervention being described. **Reviewers** – use ‘?’ if information about the element is not reported/not sufficiently reported.

† If the information is not provided in the primary paper, give details of where this information is available. This may include locations such as a published protocol or other published papers (provide citation details) or a website (provide the URL).

ǂ If completing the TIDieR checklist for a protocol, these items are not relevant to the protocol and cannot be described until the study is complete.

* We strongly recommend using this checklist in conjunction with the TIDieR guide (see *BMJ* 2014;348:g1687) which contains an explanation and elaboration for each item.

***Table S5.*** *Study: McGowan T, Ong T, Kumar A, Lunt E, Sahota O. The effect of chair-based pedal exercises for older people admitted to an acute hospital compared to standard care: a feasibility study. Age and ageing. 2018;47(3):483-6*

| **Item number** | **Item** | **Where located **** |
| --- | --- | --- |
|  |  | Primary paper (page or appendix number) |
|  | **BRIEF NAME** |  |
| **1.** | Provide the name or a phrase that describes the intervention. | Pedal exercise (PE) (p. 2)  Chair based exercises offer a simple method of improving physical activity (p. 1)  Able 2-pedal exerciser with pedometer (p. 2)  The ward team facilitates and reminds patients about exercises (p. 2)  Unsupervised (p. 2)  The ward team asked the participants once to perform exercises (p. 2)  In-hospital (p. 2)  Three times a day for 5 minutes each session, 7 days a week (p. 2)  Not described  Not described  Not described  The median time spent on the pedal exerciser was 5.08 minutes (interquartile range: 2.03-20.05) across the whole study period |
|  | **WHY** |  |
| **2.** | Describe any rationale, theory, or goal of the elements essential to the intervention. |  |
|  | **WHAT** |  |
| **3.** | Materials: Describe any physical or informational materials used in the intervention, including those provided to participants or used in intervention delivery or in training of intervention providers. Provide information on where the materials can be accessed (e.g. online appendix, URL). |  |
| **4.** | Procedures: Describe each of the procedures, activities, and/or processes used in the intervention, including any enabling or support activities. |  |
|  | **WHO PROVIDED** |  |
| **5.** | For each category of intervention provider (e.g. psychologist, nursing assistant), describe their expertise, background and any specific training given. |  |
|  | **HOW** |  |
| **6.** | Describe the modes of delivery (e.g. face-to-face or by some other mechanism, such as internet or telephone) of the intervention and whether it was provided individually or in a group. |  |
|  | **WHERE** |  |
| **7.** | Describe the type(s) of location(s) where the intervention occurred, including any necessary infrastructure or relevant features. |  |
|  | **WHEN and HOW MUCH** |  |
| **8.** | Describe the number of times the intervention was delivered and over what period of time including the number of sessions, their schedule, and their duration, intensity or dose. |  |
|  | **TAILORING** |  |
| **9.** | If the intervention was planned to be personalised, titrated or adapted, then describe what, why, when, and how. |  |
|  | **MODIFICATIONS** |  |
| **10.^ǂ^** | If the intervention was modified during the course of the study, describe the changes (what, why, when, and how). |  |
|  | **HOW WELL** |  |
| **11.** | Planned: If intervention adherence or fidelity was assessed, describe how and by whom, and if any strategies were used to maintain or improve fidelity, describe them. |  |
| **12.^ǂ^** | Actual: If intervention adherence or fidelity was assessed, describe the extent to which the intervention was delivered as planned. |  |

** **Authors** - use N/A if an item is not applicable for the intervention being described. **Reviewers** – use ‘?’ if information about the element is not reported/not sufficiently reported.

† If the information is not provided in the primary paper, give details of where this information is available. This may include locations such as a published protocol or other published papers (provide citation details) or a website (provide the URL).

ǂ If completing the TIDieR checklist for a protocol, these items are not relevant to the protocol and cannot be described until the study is complete.

* We strongly recommend using this checklist in conjunction with the TIDieR guide (see *BMJ* 2014;348:g1687) which contains an explanation and elaboration for each item.

Revised Cochrane risk-of-bias tool for randomized trials (RoB 2)

COMPLETED TEMPLATES

Edited by Julian PT Higgins, Jelena Savović, Matthew J Page, Jonathan AC Sterne
on behalf of the RoB2 Development Group

**Version of 22 August 2019**

The development of the RoB 2 tool was supported by the MRC Network of Hubs for Trials Methodology Research (MR/L004933/2- N61), with the support of the host MRC ConDuCT-II Hub (Collaboration and innovation for Difficult and Complex randomised controlled Trials In Invasive procedures - MR/K025643/1), by MRC research grant MR/M025209/1, and by a grant from The Cochrane Collaboration.


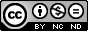


This work is licensed under a [Creative Commons Attribution-NonCommercial-NoDerivatives 4.0 International License](http://creativecommons.org/licenses/by-nc-nd/4.0/).

| ***Table S6****. Completed Risk of Bias-2 assessment of Brown et al.*  **Study details**   \| **Reference** \| Brown et al. \| \| --- \| --- \|   **Study design**   \| X \| Individually-randomized parallel-group trial \| \| --- \| --- \| \| □ \| Cluster-randomized parallel-group trial \| \| □ \| Individually randomized cross-over (or other matched) trial \|   **For the purposes of this assessment, the interventions being compared are defined as**   \| Experimental: \| Ambulation protocol and behavioral strategy \| Comparator: \| Usual care \| \| --- \| --- \| --- \| --- \|  \| **Specify which outcomes are being assessed for risk of bias** \| Reported falls, ADL-activity, community mobility, time spent out of bed \| \| --- \| --- \|  \| **Specify the numerical result being assessed.** In case of multiple alternative analyses being presented, specify the numeric result (e.g. RR = 1.52 (95% CI 0.83 to 2.77) and/or a reference (e.g. to a table, figure or paragraph) that uniquely defines the result being assessed. \| Falls: 3 versus 0. ADL-activity: 8.7 (SD: 0.3), 8.0 (SD: 0.3), *.4 (0.3), 8.1 (SD: 0.3). Community mobility: 51.5 (SD: 3.0), 41.8 (SD: 3.2), 54.0 (SD: 4.2), 52.6 (SD: 4.4). \| \| --- \| --- \|   **Is the review team’s aim for this result…?**   \| 🞭 \| to assess the effect of *assignment to intervention* (the ‘intention-to-treat’ effect) \| \| --- \| --- \| \| □ \| to assess the effect of *adhering to intervention* (the ‘per-protocol’ effect) \|   **If the aim is to assess the effect of *adhering to intervention***, select the deviations from intended intervention that should be addressed (at least one must be checked):  □ occurrence of non-protocol interventions  □ failures in implementing the intervention that could have affected the outcome  □ non-adherence to their assigned intervention by trial participants  **Which of the following sources were obtained to help inform the risk-of-bias assessment? (tick as many as apply)**  🞮 Journal article(s) with results of the trial  🞮 Trial protocol  □ Statistical analysis plan (SAP)  □ Non-commercial trial registry record (e.g. ClinicalTrials.gov record)  □ Company-owned trial registry record (e.g. GSK Clinical Study Register record)  □ “Grey literature” (e.g. unpublished thesis)  □ Conference abstract(s) about the trial  □ Regulatory document (e.g. Clinical Study Report, Drug Approval Package)  □ Research ethics application  □ Grant database summary (e.g. NIH RePORTER or Research Councils UK Gateway to Research)  □ Personal communication with trialist  □ Personal communication with the sponsor |
| --- | --- | --- | --- | --- | --- | --- | --- | --- | --- | --- | --- | --- | --- | --- | --- | --- | --- | --- | --- | --- |

## Risk of bias assessment

Responses underlined in green are potential markers for low risk of bias, and responses in red are potential markers for a risk of bias. Where questions relate only to sign posts to other questions, no formatting is used.

**Domain 1: Risk of bias arising from the randomization process**

| **Signalling questions** | **Comments** | **Response options** |
| --- | --- | --- |
| **1.1 Was the allocation sequence random?** | Quote: ‘patients were randomized to the MP or UC group using a block randomization strategy where for every block of 10, a total of 5 were allocated to each study arm.  Quote: To achieve allocation concealment, sequentially sealed envelopes with assignments based  on the randomization schedule were used. | Y |
| **1.2 Was the allocation sequence concealed until participants were enrolled and assigned to interventions?** |  | Y |
| **1.3 Did baseline differences between intervention groups suggest a problem with the randomization process?** | No statistically significant differences between baseline characteristics. No relevant baseline differences mentioned by authors. | N |
| **Risk-of-bias judgement** |  | Low |
| Optional: What is the predicted direction of bias arising from the randomization process? |  | NA |

Domain 2: Risk of bias due to deviations from the intended interventions (*effect of assignment to intervention*)

| **Signalling questions** | **Comments** | **Response options** |
| --- | --- | --- |
| **2.1. Were participants aware of their assigned intervention during the trial?** | Quote: All members of the research team who transferred and walked with patients received in-depth training in safe patient handling techniques by BVAMC physical therapists.  Comment: Probably both personnel and participants aware for intended intervention.. | PY |
| **2.2. Were carers and people delivering the interventions aware of participants' assigned intervention during the trial?** |  | PY |
| **2.3. If Y/PY/NI to 2.1 or 2.2: Were there deviations from the intended intervention that arose because of the trial context?** | The authors reported no deviations from the intended intervention. The protocol presents the same intervention as the article. | PN |
| **2.4 If Y/PY to 2.3: Were these deviations likely to have affected the outcome?** |  | NA |
| **2.5. If Y/PY/NI to 2.4: Were these deviations from intended intervention balanced between groups?** |  | NA |
| **2.6 Was an appropriate analysis used to estimate the effect of assignment to intervention?** |  | PY |
| **2.7 If N/PN/NI to 2.6: Was there potential for a substantial impact (on the result) of the failure to analyse participants in the group to which they were randomized?** |  | NA |
| **Risk-of-bias judgement** |  | Some concerns |
| Optional: What is the predicted direction of bias due to deviations from intended interventions? |  | Towards null |

Domain 3: Missing outcome data

| **Signalling questions** | **Comments** | **Response options** |
| --- | --- | --- |
| **3.1 Were data for this outcome available for all, or nearly all, participants randomized?** | Quote: Because more patients in the MP group (n = 6) than the UC group (n = 2) were withdrawn because of death or ICU transfer before study end, there is the potential for biased results….‘‘The sensitivity analysis suggests that missing values had little effect on group differences in LSA scores because results of complete case analysis were similar to those derived from replacing missing data with imputed values.’  Comment: More participants dropped out from the MP. The authors conducted some appropriate analysis which showed that these differences most likely didn’t influence the outcome data. | Y |
| **3.2 If N/PN/NI to 3.1: Is there evidence that the result was not biased by missing outcome data?** |  | NA |
| **3.3 If N/PN to 3.2: Could missingness in the outcome depend on its true value?** |  | NA |
| **3.4 If Y/PY/NI to 3.3: Is it likely that missingness in the outcome depended on its true value?** |  | NA |
| **Risk-of-bias judgement** |  | Low |
| Optional: What is the predicted direction of bias due to missing outcome data? |  | NA |

Domain 4: Risk of bias in measurement of the outcome

| **Signalling questions** | **Comments** | **Response options** |
| --- | --- | --- |
| **4.1 Was the method of measuring the outcome inappropriate?** | Quote: After randomization, assessments were completed throughout the hospitalization by a separate group of masked assessors.  All used assessments are well supported by evidence. However, the technical failure of accelerometers was unfortunate. | N |
| **4.2 Could measurement or ascertainment of the outcome have differed between intervention groups?** |  | PN |
| **4.3 If N/PN/NI to 4.1 and 4.2: Were outcome assessors aware of the intervention received by study participants?** | Quote: After randomization, assessments were completed throughout the hospitalization by a separate group of masked assessors. | N |
| **4.4 If Y/PY/NI to 4.3: Could assessment of the outcome have been influenced by knowledge of intervention received?** |  | NA |
| **4.5 If Y/PY/NI to 4.4:** **Is it likely that assessment of the outcome was influenced by knowledge of intervention received?** |  | NA |
| **Risk-of-bias judgement** |  | Low |
| Optional: What is the predicted direction of bias in measurement of the outcome? |  | NA |

Domain 5: Risk of bias in selection of the reported result

| **Signalling questions** | **Comments** | **Response options** |
| --- | --- | --- |
| **5.1 Were the data that produced this result analysed in accordance with a pre-specified analysis plan that was finalized before unblinded outcome data were available for analysis?** | Our initial trial registration described time out of bed using accelerometers as our primary outcome.29 However, technical failures in this measure precluded use of accelerometer data.  Therefore, this report focuses on outcomes from other aims in the protocol. | Y |
| **Is the numerical result being assessed likely to have been selected, on the basis of the results, from...** |  |  |
| **5.2. ... multiple eligible outcome measurements (e.g. scales, definitions, time points) within the outcome domain?** | Standardized, accessible outcomes | N |
| **5.3 ... multiple eligible analyses of the data?** | Standard, straightforward analyses. However, the analysis did not include within-group changes. | N |
| **Risk-of-bias judgement** | The authors performed no overall analysis for assignment to interventions. The effect of assignment has been tested per follw-up (t0, t1, etc.). In addition, the authors did not analyse change-scores (from t0 to t1) but compared differences between groups (intervention t0 versus usual care t0). | Some concerns |
| Optional: What is the predicted direction of bias due to selection of the reported result? |  | Towards null |

Overall risk of bias

| **Risk-of-bias judgement** |  | Some concerns |
| --- | --- | --- |
| Optional: What is the overall predicted direction of bias for this outcome? |  | Towards null |


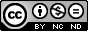


This work is licensed under a [Creative Commons Attribution-NonCommercial-NoDerivatives 4.0 International License](http://creativecommons.org/licenses/by-nc-nd/4.0/).

| ***Table S7****. Completed Risk of Bias-2 assessment of Dall et al.*  **Study details**   \| **Reference** \| Dall et al. \| \| --- \| --- \|   **Study design**   \| X \| Individually-randomized parallel-group trial \| \| --- \| --- \| \| □ \| Cluster-randomized parallel-group trial \| \| □ \| Individually randomized cross-over (or other matched) trial \|   **For the purposes of this assessment, the interventions being compared are defined as**   \| Experimental: \| Visual feedback with a tablet \| Comparator: \| Without feedback \| \| --- \| --- \| --- \| --- \|  \| **Specify which outcomes are being assessed for risk of bias** \| Reported falls, time spent lying-sitting-standing-walking \| \| --- \| --- \|  \| **Specify the numerical result being assessed.** In case of multiple alternative analyses being presented, specify the numeric result (e.g. RR = 1.52 (95% CI 0.83 to 2.77) and/or a reference (e.g. to a table, figure or paragraph) that uniquely defines the result being assessed. \| Reported falls: 0 versus 0. Time spent lying in minutes per day. \| \| --- \| --- \|   **Is the review team’s aim for this result…?**   \| 🞮 \| to assess the effect of *assignment to intervention* (the ‘intention-to-treat’ effect) \| \| --- \| --- \| \| □ \| to assess the effect of *adhering to intervention* (the ‘per-protocol’ effect) \|   **If the aim is to assess the effect of *adhering to intervention***, select the deviations from intended intervention that should be addressed (at least one must be checked):  □ occurrence of non-protocol interventions  □ failures in implementing the intervention that could have affected the outcome  □ non-adherence to their assigned intervention by trial participants  **Which of the following sources were obtained to help inform the risk-of-bias assessment? (tick as many as apply)**  🞮 Journal article(s) with results of the trial  □ Trial protocol  □ Statistical analysis plan (SAP)  □ Non-commercial trial registry record (e.g. ClinicalTrials.gov record)  □ Company-owned trial registry record (e.g. GSK Clinical Study Register record)  □ “Grey literature” (e.g. unpublished thesis)  □ Conference abstract(s) about the trial  □ Regulatory document (e.g. Clinical Study Report, Drug Approval Package)  □ Research ethics application  □ Grant database summary (e.g. NIH RePORTER or Research Councils UK Gateway to Research)  □ Personal communication with trialist  □ Personal communication with the sponsor |
| --- | --- | --- | --- | --- | --- | --- | --- | --- | --- | --- | --- | --- | --- | --- | --- | --- | --- | --- | --- | --- |

## Risk of bias assessment

Responses underlined in green are potential markers for low risk of bias, and responses in red are potential markers for a risk of bias. Where questions relate only to sign posts to other questions, no formatting is used.

**Domain 1: Risk of bias arising from the randomization process**

| **Signalling questions** | **Comments** | **Response options** |
| --- | --- | --- |
| **1.1 Was the allocation sequence random?** | Quote: Participants were assigned to the cohorts in order of admission to the ward; i.e. the ﬁrst 18 included patients were assigned to cohort one, the next 18 to cohort two and so on.  Quote: “As this was an “open-label” study neither the participants nor the clinical staﬀ were blinded to treatment allocation.’ | N |
| **1.2 Was the allocation sequence concealed until participants were enrolled and assigned to interventions?** |  | N |
| **1.3 Did baseline differences between intervention groups suggest a problem with the randomization process?** | No statistically significant differences between baseline characteristics. No relevant baseline differences mentioned by authors. | PN |
| **Risk-of-bias judgement** |  | High |
| Optional: What is the predicted direction of bias arising from the randomization process? |  | Favours experimental |

Domain 2: Risk of bias due to deviations from the intended interventions (*effect of assignment to intervention*)

| **Signalling questions** | **Comments** | **Response options** |
| --- | --- | --- |
| **2.1. Were participants aware of their assigned intervention during the trial?** | Quote: “As this was an “open-label” study neither the participants nor the clinical staﬀ were blinded to treatment allocation.’ | Y |
| **2.2. Were carers and people delivering the interventions aware of participants' assigned intervention during the trial?** |  | Y |
| **2.3. If Y/PY/NI to 2.1 or 2.2: Were there deviations from the intended intervention that arose because of the trial context?** | The authors reported no deviations from the intended intervention. The protocol presents the same intervention as the article. | PN |
| **2.4 If Y/PY to 2.3: Were these deviations likely to have affected the outcome?** |  | NA |
| **2.5. If Y/PY/NI to 2.4: Were these deviations from intended intervention balanced between groups?** |  | NA |
| **2.6 Was an appropriate analysis used to estimate the effect of assignment to intervention?** | The analyses with unpaired t-tests and ANCOVA seem appropriate. | Y |
| **2.7 If N/PN/NI to 2.6: Was there potential for a substantial impact (on the result) of the failure to analyse participants in the group to which they were randomized?** |  | NA |
| **Risk-of-bias judgement** |  | Some concerns |
| Optional: What is the predicted direction of bias due to deviations from intended interventions? | Open label use of intervention might favour experimental group. For example, healthcare professionals and family might stimulate patients more often as a result of the presence of a tablet (intended versus unintended use). | Favours experimental |

Domain 3: Missing outcome data

| **Signalling questions** | **Comments** | **Response options** |
| --- | --- | --- |
| **3.1 Were data for this outcome available for all, or nearly all, participants randomized?** | “Upon data analysis, 25 in feedback group and 19 in no feedback group were excluded due to recordings of<24h” 🡪 Were equally distributed in both groups. (Reasons for missing outcome data unlikely to be related to true outcome & Missing outcome data balanced in numbers across intervention groups, with similar reasons for missing data across groups.) | PY |
| **3.2 If N/PN/NI to 3.1: Is there evidence that the result was not biased by missing outcome data?** |  | NA |
| **3.3 If N/PN to 3.2: Could missingness in the outcome depend on its true value?** |  | NA |
| **3.4 If Y/PY/NI to 3.3: Is it likely that missingness in the outcome depended on its true value?** |  | NA |
| **Risk-of-bias judgement** |  | Low |
| Optional: What is the predicted direction of bias due to missing outcome data? |  | NA |

Domain 4: Risk of bias in measurement of the outcome

| **Signalling questions** | **Comments** | **Response options** |
| --- | --- | --- |
| **4.1 Was the method of measuring the outcome inappropriate?** | The quality of accelerometry has not been reported. The falls are well-reported. | PY |
| **4.2 Could measurement or ascertainment of the outcome have differed between intervention groups?** | Same methods between groups. | N |
| **4.3 If N/PN/NI to 4.1 and 4.2: Were outcome assessors aware of the intervention received by study participants?** | Physical therapists took care for both the intervention, as the outcome measurements. Quote: ‘The physiotherapists mounted the accelerometers, and if appropriate according to allocation, installed feedback screens on the bedside table’ | Y |
| **4.4 If Y/PY/NI to 4.3: Could assessment of the outcome have been influenced by knowledge of intervention received?** | Reporting of time spent lying-sitting-standing-walking by hardware and software, no human interactions. Reported falls were well-reported. | PN |
| **4.5 If Y/PY/NI to 4.4: Is it likely that assessment of the outcome was influenced by knowledge of intervention received?** |  | PN |
| **Risk-of-bias judgement** |  | High |
| Optional: What is the predicted direction of bias in measurement of the outcome? |  | Towards null |

Domain 5: Risk of bias in selection of the reported result

| **Signalling questions** | **Comments** | **Response options** |
| --- | --- | --- |
| **5.1 Were the data that produced this result analysed in accordance with a pre-specified analysis plan that was finalized before unblinded outcome data were available for analysis?** | The study protocol is not available but it is clear that the published reports include all expected outcomes, including those that were prespecified (convincing text of this nature may be uncommon). | PY |
| **Is the numerical result being assessed likely to have been selected, on the basis of the results, from...** |  |  |
| **5.2. ... multiple eligible outcome measurements (e.g. scales, definitions, time points) within the outcome domain?** |  | PN |
| **5.3 ... multiple eligible analyses of the data?** |  | N |
| **Risk-of-bias judgement** |  | Low |
| Optional: What is the predicted direction of bias due to selection of the reported result? |  | NA |

Overall risk of bias

| **Risk-of-bias judgement** |  | High |
| --- | --- | --- |
| Optional: What is the overall expected direction of bias for this outcome? | The risk of bias arising from randomization and (some concerns) for deviation of interventions might favour the experimental group. | Favours experimental |


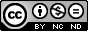


This work is licensed under a [Creative Commons Attribution-NonCommercial-NoDerivatives 4.0 International License](http://creativecommons.org/licenses/by-nc-nd/4.0/).

| ***Table S8****. Completed Risk of Bias-2 assessment of Killey and Watt.*  **Study details**   \| **Reference** \| Killey and Watt \| \| --- \| --- \|   **Study design**   \| X \| Individually-randomized parallel-group trial \| \| --- \| --- \| \| □ \| Cluster-randomized parallel-group trial \| \| □ \| Individually randomized cross-over (or other matched) trial \|   **For the purposes of this assessment, the interventions being compared are defined as**   \| Experimental: \| Twice-daily extra walking \| Comparator: \| Standard nursing care \| \| --- \| --- \| --- \| --- \|  \| **Specify which outcomes are being assessed for risk of bias** \| Reported falls, ADL-activity and hospital mobility \| \| --- \| --- \|  \| **Specify the numerical result being assessed.** In case of multiple alternative analyses being presented, specify the numeric result (e.g. RR = 1.52 (95% CI 0.83 to 2.77) and/or a reference (e.g. to a table, figure or paragraph) that uniquely defines the result being assessed. \| Reported falls: 2 versus 0. ADL activity in performance score (mean, SD). Hospital mobility in meters (mean, SD). \| \| --- \| --- \|   **Is the review team’s aim for this result…?**   \| 🞮 \| to assess the effect of *assignment to intervention* (the ‘intention-to-treat’ effect) \| \| --- \| --- \| \| □ \| to assess the effect of *adhering to intervention* (the ‘per-protocol’ effect) \|   **If the aim is to assess the effect of *adhering to intervention***, select the deviations from intended intervention that should be addressed (at least one must be checked):  □ occurrence of non-protocol interventions  □ failures in implementing the intervention that could have affected the outcome  □ non-adherence to their assigned intervention by trial participants  **Which of the following sources were obtained to help inform the risk-of-bias assessment? (tick as many as apply)**  🞮 Journal article(s) with results of the trial  □ Trial protocol  □ Statistical analysis plan (SAP)  □ Non-commercial trial registry record (e.g. ClinicalTrials.gov record)  □ Company-owned trial registry record (e.g. GSK Clinical Study Register record)  □ “Grey literature” (e.g. unpublished thesis)  □ Conference abstract(s) about the trial  □ Regulatory document (e.g. Clinical Study Report, Drug Approval Package)  □ Research ethics application  □ Grant database summary (e.g. NIH RePORTER or Research Councils UK Gateway to Research)  □ Personal communication with trialist  □ Personal communication with the sponsor |
| --- | --- | --- | --- | --- | --- | --- | --- | --- | --- | --- | --- | --- | --- | --- | --- | --- | --- | --- | --- | --- |

## Risk of bias assessment

Responses underlined in green are potential markers for low risk of bias, and responses in red are potential markers for a risk of bias. Where questions relate only to sign posts to other questions, no formatting is used.

**Domain 1: Risk of bias arising from the randomization process**

| **Signalling questions** | **Comments** | **Response options** |
| --- | --- | --- |
| **1.1 Was the allocation sequence random?** | Quote: The use of a true randomisation process was not possible in this study due to time and resource constraints. Therefore a convenience sample of those patients who met the sample criteria and who consented to participate were allocated consecutively in order of admission to a particular unit  Quote: Due to limited resources, the researchers were required to perform every part of the process except for the administration of the walking and so it was not possible to systematically blind the researchers as to who was in which group. | N |
| **1.2 Was the allocation sequence concealed until participants were enrolled and assigned to interventions?** |  | N |
| **1.3 Did baseline differences between intervention groups suggest a problem with the randomization process?** | No information available on baseline characteristics. The authors reported no relevant differences between groups. | PN |
| **Risk-of-bias judgement** |  | High |
| Optional: What is the predicted direction of bias arising from the randomization process? |  | Towards null |

Domain 2: Risk of bias due to deviations from the intended interventions (*effect of assignment to intervention*)

| **Signalling questions** | **Comments** | **Response options** |
| --- | --- | --- |
| **2.1. Were participants aware of their assigned intervention during the trial?** | Comment: Whether the participants were aware of the contents of this study is not described. Probably blinding is not done.  Comment: The researchers did not administrate the extra walking, but the staff is aware of the intervention. | PY |
| **2.2. Were carers and people delivering the interventions aware of participants' assigned intervention during the trial?** |  | PY |
| **2.3. If Y/PY/NI to 2.1 or 2.2: Were there deviations from the intended intervention that arose because of the trial context?** | The authors reported no deviations from the intended intervention. | PN |
| **2.4 If Y/PY to 2.3: Were these deviations likely to have affected the outcome?** |  | NA |
| **2.5. If Y/PY/NI to 2.4: Were these deviations from intended intervention balanced between groups?** |  | NA |
| **2.6 Was an appropriate analysis used to estimate the effect of assignment to intervention?** | The analysis did not control for between-group differences in baseline characteristics. In addition, the authors did not correct for within-group changes and tested only between-group differences per follow-up. | PN |
| **2.7 If N/PN/NI to 2.6: Was there potential for a substantial impact (on the result) of the failure to analyse participants in the group to which they were randomized?** |  | PN |
| **Risk-of-bias judgement** |  | Some concerns |
| Optional: What is the predicted direction of bias due to deviations from intended interventions? |  | Towards null |

Domain 3: Missing outcome data

| **Signalling questions** | **Comments** | **Response options** |
| --- | --- | --- |
| **3.1 Were data for this outcome available for all, or nearly all, participants randomized?** | Comment: One person in the control group was overruled by the doctor (selection bias). Furthermore, no intention to treat analysis were used.  Reasons for not completing the study for both groups are not explained. The groups differed on the SEE-scale, which could potentially influence the outcome, as SEE is an important predictor for patient mobility. | N |
| **3.2 If N/PN/NI to 3.1: Is there evidence that the result was not biased by missing outcome data?** |  | N |
| **3.3 If N/PN to 3.2: Could missingness in the outcome depend on its true value?** | Quote: 2 patients were later excluded because they completed less than 70% of their walks. Statistically significant differences between non-completers and completers were found. | Y |
| **3.4 If Y/PY/NI to 3.3: Is it likely that missingness in the outcome depended on its true value?** |  | Y |
| **Risk-of-bias judgement** |  | High |
| Optional: What is the predicted direction of bias due to missing outcome data? |  | Unpredictable |

Domain 4: Risk of bias in measurement of the outcome

| **Signalling questions** | **Comments** | **Response options** |
| --- | --- | --- |
| **4.1 Was the method of measuring the outcome inappropriate?** | Adequate methods | N |
| **4.2 Could measurement or ascertainment of the outcome have differed between intervention groups?** |  | N |
| **4.3 If N/PN/NI to 4.1 and 4.2: Were outcome assessors aware of the intervention received by study participants?** |  | Y |
| **4.4 If Y/PY/NI to 4.3: Could assessment of the outcome have been influenced by knowledge of intervention received?** |  | PN |
| **4.5 If Y/PY/NI to 4.4: Is it likely that assessment of the outcome was influenced by knowledge of intervention received?** |  | PN |
| **Risk-of-bias judgement** |  | Low |
| Optional: What is the predicted direction of bias in measurement of the outcome? |  | NA |

Domain 5: Risk of bias in selection of the reported result

| **Signalling questions** | **Comments** | **Response options** |
| --- | --- | --- |
| **5.1 Were the data that produced this result analysed in accordance with a pre-specified analysis plan that was finalized before unblinded outcome data were available for analysis?** | Protocol not available | NI |
| **Is the numerical result being assessed likely to have been selected, on the basis of the results, from...** |  |  |
| **5.2. ... multiple eligible outcome measurements (e.g. scales, definitions, time points) within the outcome domain?** | Single, straightforward outcomes | N |
| **5.3 ... multiple eligible analyses of the data?** | Single, straightforward outcomes | N |
| **Risk-of-bias judgement** |  | Some concerns |
| Optional: What is the predicted direction of bias due to selection of the reported result? |  | Unpredictable |

Overall risk of bias

| **Risk-of-bias judgement** |  | High |
| --- | --- | --- |
| Optional: What is the overall predicted direction of bias for this outcome? |  | Towards null |


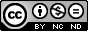


This work is licensed under a [Creative Commons Attribution-NonCommercial-NoDerivatives 4.0 International License](http://creativecommons.org/licenses/by-nc-nd/4.0/).

| ***Table S9****. Completed Risk of Bias-2 assessment of McGowan et al.*  **Study details**   \| **Reference** \| McGowan et al. \| \| --- \| --- \|   **Study design**   \| X \| Individually-randomized parallel-group trial \| \| --- \| --- \| \| □ \| Cluster-randomized parallel-group trial \| \| □ \| Individually randomized cross-over (or other matched) trial \|   **For the purposes of this assessment, the interventions being compared are defined as**   \| Experimental: \| Pedal exercises \| Comparator: \| Standard care \| \| --- \| --- \| --- \| --- \|  \| **Specify which outcomes are being assessed for risk of bias** \| ADL-activity, time spent standing-walking, revolutions cycled, pedal exercises. \| \| --- \| --- \|  \| **Specify the numerical result being assessed.** In case of multiple alternative analyses being presented, specify the numeric result (e.g. RR = 1.52 (95% CI 0.83 to 2.77) and/or a reference (e.g. to a table, figure or paragraph) that uniquely defines the result being assessed. \| ADL-activity: score (mean, SD). Time spent standing-walking: percentage per day (median, interquartile range), revolutions cycled: number (median, interquartile range), pedal exercise: minutes (mean, confidence interval). \| \| --- \| --- \|   **Is the review team’s aim for this result…?**   \| 🞮 \| to assess the effect of *assignment to intervention* (the ‘intention-to-treat’ effect) \| \| --- \| --- \| \| □ \| to assess the effect of *adhering to intervention* (the ‘per-protocol’ effect) \|   **If the aim is to assess the effect of *adhering to intervention***, select the deviations from intended intervention that should be addressed (at least one must be checked):  □ occurrence of non-protocol interventions  □ failures in implementing the intervention that could have affected the outcome  □ non-adherence to their assigned intervention by trial participants  **Which of the following sources were obtained to help inform the risk-of-bias assessment? (tick as many as apply)**  🞮 Journal article(s) with results of the trial  🞮 Trial protocol  □ Statistical analysis plan (SAP)  □ Non-commercial trial registry record (e.g. ClinicalTrials.gov record)  □ Company-owned trial registry record (e.g. GSK Clinical Study Register record)  □ “Grey literature” (e.g. unpublished thesis)  □ Conference abstract(s) about the trial  □ Regulatory document (e.g. Clinical Study Report, Drug Approval Package)  □ Research ethics application  □ Grant database summary (e.g. NIH RePORTER or Research Councils UK Gateway to Research)  □ Personal communication with trialist  □ Personal communication with the sponsor |
| --- | --- | --- | --- | --- | --- | --- | --- | --- | --- | --- | --- | --- | --- | --- | --- | --- | --- | --- | --- | --- |

## Risk of bias assessment

Responses underlined in green are potential markers for low risk of bias, and responses in red are potential markers for a risk of bias. Where questions relate only to sign posts to other questions, no formatting is used.

**Domain 1: Risk of bias arising from the randomization process**

| **Signalling questions** | **Comments** | **Response options** |
| --- | --- | --- |
| **1.1 Was the allocation sequence random?** | Quote: Randomisation was generated using an online webtool (www.randomization. com).  Quote: Randomisation sequence was only known to the chief investigator who was not involved in screening patients. | Y |
| **1.2 Was the allocation sequence concealed until participants were enrolled and assigned to interventions?** |  | Y |
| **1.3 Did baseline differences between intervention groups suggest a problem with the randomization process?** | No statistically significant differences were found between baseline characteristics of participants in the two groups. No relevant differences were reported by the authors. | N |
| **Risk-of-bias judgement** |  | Low |
| Optional: What is the predicted direction of bias arising from the randomization process? |  | NA |

Domain 2: Risk of bias due to deviations from the intended interventions (*effect of assignment to intervention*)

| **Signalling questions** | **Comments** | **Response options** |
| --- | --- | --- |
| **2.1. Were participants aware of their assigned intervention during the trial?** | Participants and personnel were not blinded, as this was not possible: Quote: “The ward team would facilitate the pedal exercises and remind patients of it” 🡪 Quote: ‘The research team visited each ward and participant allocated to the pedal exercise group daily to address any possible technical or equipment issues’  The research team was aware of the kind of treatment patients received. | Y |
| **2.2. Were carers and people delivering the interventions aware of participants' assigned intervention during the trial?** |  | Y |
| **2.3. If Y/PY/NI to 2.1 or 2.2: Were there deviations from the intended intervention that arose because of the trial context?** | The authors reported no deviations from the intended intervention. The intervention is likely to be delivered as planned. | PN |
| **2.4 If Y/PY to 2.3: Were these deviations likely to have affected the outcome?** |  | NA |
| **2.5. If Y/PY/NI to 2.4: Were these deviations from intended intervention balanced between groups?** |  | NA |
| **2.6 Was an appropriate analysis used to estimate the effect of assignment to intervention?** | The effect of assignment was not estimated. | N |
| **2.7 If N/PN/NI to 2.6: Was there potential for a substantial impact (on the result) of the failure to analyse participants in the group to which they were randomized?** |  | PN |
| **Risk-of-bias judgement** |  | Some concerns |
| Optional: What is the predicted direction of bias due to deviations from intended interventions? |  | Towards null |

Domain 3: Missing outcome data

| **Signalling questions** | **Comments** | **Response options** |
| --- | --- | --- |
| **3.1 Were data for this outcome available for all, or nearly all, participants randomized?** | Two patients were excluded from analysis. | N |
| **3.2 If N/PN/NI to 3.1: Is there evidence that the result was not biased by missing outcome data?** | No data collected by accelerometer. | PY |
| **3.3 If N/PN to 3.2: Could missingness in the outcome depend on its true value?** |  | NA |
| **3.4 If Y/PY/NI to 3.3: Is it likely that missingness in the outcome depended on its true value?** |  | NA |
| **Risk-of-bias judgement** | The original protocol and goal of the study were changed from a feasibility study to an intervention study. | High |
| Optional: What is the predicted direction of bias due to missing outcome data? |  | Unpredictable |

Domain 4: Risk of bias in measurement of the outcome

| **Signalling questions** | **Comments** | **Response options** |
| --- | --- | --- |
| **4.1 Was the method of measuring the outcome inappropriate?** | The quality and validity of the accelerometer has not been reported. Other methods are valid and common for use in this population for this purpose. | PN |
| **4.2 Could measurement or ascertainment of the outcome have differed between intervention groups?** |  | PN |
| **4.3 If N/PN/NI to 4.1 and 4.2: Were outcome assessors aware of the intervention received by study participants?** | The research team was aware of the kind of treatment patients received. | Y |
| **4.4 If Y/PY/NI to 4.3: Could assessment of the outcome have been influenced by knowledge of intervention received?** | ADL-activity was self-reported and might have been influenced. Time spent standing-walking, revolutions cycled and pedal exercising time were collected with hardware and analysed with standard software. | PN |
| **4.5 If Y/PY/NI to 4.4: Is it likely that assessment of the outcome was influenced by knowledge of intervention received?** |  | PN |
| **Risk-of-bias judgement** |  | Low |
| Optional: What is the predicted direction of bias in measurement of the outcome? |  | NA |

Domain 5: Risk of bias in selection of the reported result

| **Signalling questions** | **Comments** | **Response options** |
| --- | --- | --- |
| **5.1 Were the data that produced this result analysed in accordance with a pre-specified analysis plan that was finalized before unblinded outcome data were available for analysis?** | The analysis followed the plan reported in the trial protocol. However, the differences between groups were not tested for statistical significance. Only within-group differences were reported and compared. | PY |
| **Is the numerical result being assessed likely to have been selected, on the basis of the results, from...** |  |  |
| **5.2. ... multiple eligible outcome measurements (e.g. scales, definitions, time points) within the outcome domain?** | Single, straightforward outcomes and analysis | N |
| **5.3 ... multiple eligible analyses of the data?** | Single, straightforward outcomes and analysis | N |
| **Risk-of-bias judgement** |  | Some concerns |
| Optional: What is the predicted direction of bias due to selection of the reported result? |  | NA |

Overall risk of bias

| **Risk-of-bias judgement** |  | High |
| --- | --- | --- |
| Optional: What is the overall predicted direction of bias for this outcome? |  | Towards null |


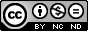


This work is licensed under a [Creative Commons Attribution-NonCommercial-NoDerivatives 4.0 International License](http://creativecommons.org/licenses/by-nc-nd/4.0/).
