# Supplementary figures and images for: Effects of General Physical Activity Promoting Interventions on Functional Outcomes in Patients Hospitalized over 48 Hours: A Systematic Review and Meta-Analysis of Randomized Controlled Trials
Source: Int J Environ Res Public Health. 2021 Jan 29;18(3):1233. doi: 10.3390/ijerph18031233 (PMC7908565; doi:10.3390/ijerph18031233)

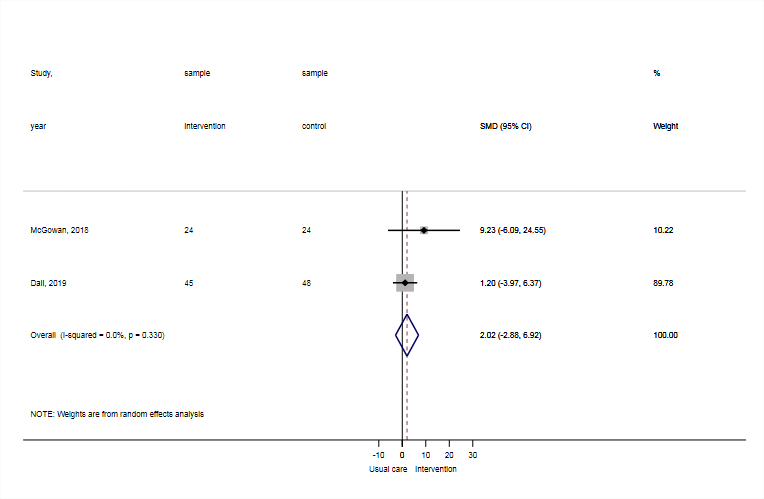

Supplement: Supplementary file 1 [file ijerph-18-01233-s001.zip › Figure 3 Time spent standing and walking.tif]

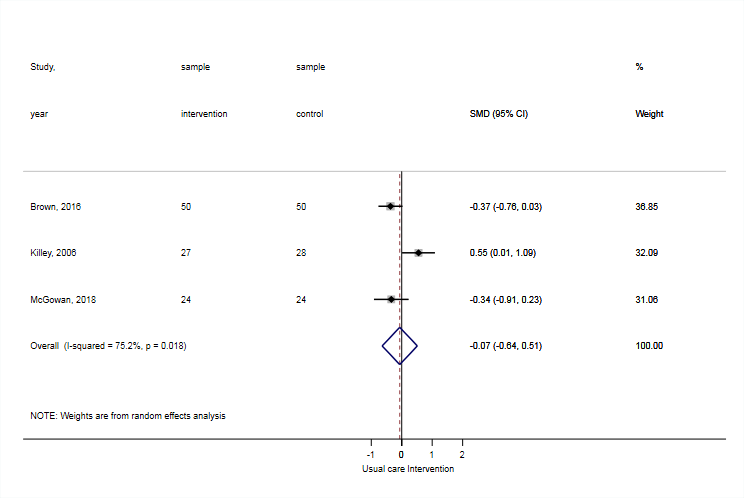

Supplement: Supplementary file 1 [file ijerph-18-01233-s001.zip › Figure 2 ADL-activity performance.tif]
